# Development and Evaluation of a Pioneer School-Based Gifted Education Program (Project GIFT) for Primary and Secondary Students in Hong Kong

**DOI:** 10.3390/ijerph19084832

**Published:** 2022-04-15

**Authors:** Daniel Tan Lei Shek, Alan Chi Keung Cheung, Anna Na Na Hui, Kim Hung Leung, Ruby Shui Ha Cheung

**Affiliations:** 1 Department of Applied Social Sciences, The Hong Kong Polytechnic University, Hong Kong; wlkh22@yahoo.com.hk; 2 Department of Educational Administration and Policy, Faculty of Education, The Chinese University of Hong Kong, Hong Kong; alancheung@cuhk.edu.hk; 3 Department of Social and Behavioural Sciences, City University of Hong Kong, Hong Kong; annahui@cityu.edu.hk; 4 Hong Kong Baptist University Affiliated School Wong Kam Fai Secondary and Primary School, Hong Kong; rshcheung@hkbuas.edu.hk

**Keywords:** gifted education, Hong Kong, development, evaluation

## Abstract

In this study, we used a quasi-experimental research design with pretest and post-test data collected from an experimental group and a control group to investigate changes in students after participating in a school-based gifted education program (Project GIFT) in Hong Kong. There were 3207 successfully matched students (3rd to 9th graders) joining the Level 1 program (for all students) alone or both the Level 1 program and Level 2 program (for gifted students). Participants of the experimental and control groups completed validated measures on creativity, multiple intelligences, gifted characteristics, self-efficacy, psychological well-being, and satisfaction with life before and after participating in the program(s). One-way ANCOVA results revealed that students in the experimental groups showed positive changes after joining the program(s), with a greater impact for students joining both Level 1 and Level 2 programs. Students participating in both Level 1 and Level 2 programs displayed significant improvement in creativity, academic performance, logical–mathematical intelligence, intrapersonal intelligence, self-efficacy, autonomy, environmental mastery, and personal growth compared to the control counterparts. This study illustrates the benefits of the Level 1 and Level 2 programs in promoting the holistic development of the program participants.

## 1. Introduction

Gifted education and talent development programs are important tools for nurturing human capital and promoting economic growth in many societies [1]. They provide opportunities for individuals to develop their talents and achieve their potential. According to Renzulli’s (1978) three-ring conception of giftedness [2], gifted behaviors include three clusters of abilities—above average ability, high level of task commitment and creativity. These clusters of abilities would emerge after providing students with a wide range of learning opportunities and services, resources, and encouragement by teachers and parents. As such, various empirically supported strategies and practices have been developed to maximize the potential of students such as acceleration (e.g., [3]), enrichment (e.g., [4]), differentiation (e.g., [5]), ability grouping (e.g., [6]), curriculum compacting (e.g., [7]), and extracurricular programs (e.g., [8]). Although the benefits of these strategies to students have been found, systematic research based on a comprehensive framework of a gifted education program with the inclusion of different effective teaching strategies to nurture gifted students is limited (see [9]). Moreover, most of the existing studies examining the effectiveness of programs aiming to nurture talents in students have been conducted predominately in Western societies. As such, this study explored the effectiveness of a school-based gifted education program in Hong Kong.

### 1.1. Gifted Education in Hong Kong

Based on the response-to-intervention (RTI) model focusing on primary, secondary and tertiary support for needy students [10], the Education Bureau of the Government of the Hong Kong Special Administrative Region has implemented a similar three-tier implementation model of gifted education to promote school-based gifted education in local primary and secondary schools. Level 1 intervention occurs at the classroom level and for all students. It utilizes pedagogies to tap students’ potentials in terms of creativity, problem-solving, critical thinking, and leadership competency within regular classroom learning and to differentiate teaching with enrichment and extension activities in different subjects. Level 2 initiatives operate through pull-out programs in subjects or interdisciplinary areas for gifted students within the school setting, with differentiated curricula and programs designed for students with specific talents, outstanding academic results, or good performance in specific non-academic domains such as creativity and leadership. Level 3 intervention caters for exceptionally gifted students by offering tailor-made off-site programs in collaboration with universities and other professional bodies. This three-tier model is similar to the three-tier implementation model for gifted education in the United States [9]. Nevertheless, in contrast to the U.S. implementation policy of gifted education that specifies that gifted education programs must be validated by research, evidence for the effectiveness of the Hong Kong three-tier implementation model of gifted education has been lacking [11].

Moreover, although all levels are equally important in the nurturing talents and capabilities of every student, particularly for the gifted and talented, only Level 3 of Hong Kong’s three-tier implementation model of gifted education has been emphasized in terms of policy initiatives [12]. In the 2006 Policy Address of Hong Kong Special Administrative Region Government, the Government planned to establish an academy for gifted education which would offer off-site programs for gifted students, teachers, and parents. As such, the Hong Kong Academy for Gifted Education (HKAGE) was set up in 2008. The Government further set up the Gifted Education Fund of HK$ 800 million to support Level 3 programs of HKAGE. To date, almost all studies about gifted education in Hong Kong have been limited to Level 3 programs (e.g., [13,14,15,16]). In contrast, research on the development and evaluation of Level 1 and Level 2 programs are almost non-existent. As research-based interventions is a vital element of an effective multi-tiered gifted education model [17], this pioneer study fills the gap by examining the development and effectiveness of Level 1 and Level 2 programs of school-based gifted education (Project GIFT) in a Chinese context. Research in the Chinese context is important because of the huge population size of Chinese people. 

### 1.2. Project GIFT in Hong Kong

In response to the paucity of research on the three-tier implementation model of gifted education in Hong Kong, the Project “Jockey Club ‘Giftedness into Flourishing Talents’ Project” (Project GIFT) was launched from 2016 to 2020. It was designed to help local schools to: (a) develop their own school-based gifted education policy; (b) enhance the strengths and capabilities, whole-person development, and well-being of “gifted” and “ordinary” students; (c) develop the professional competence of school personnel in school-based gifted education; and (d) foster parents’ capability to nurture gifted offspring. With financial support from the Hong Kong Jockey Club Charities Trust (earmarked grant of HKD 48,500,000 which is roughly equivalent to USD 6,217,949), scholars from four universities in Hong Kong formed a research team to develop curriculum-based gifted programs for students, provide professional training to teachers, organize seminars for parents and conduct an evaluation of the programs. There were 20 project schools (experimental schools) and 8 non-project schools (control schools) joining the project.

There are several conceptual pillars in the Project GIFT. First, instead of viewing IQ scores as the sole criterion to define giftedness and identify gifted students, the Project adopted a multidimensional conception of giftedness based on Gardner’s (2011) Multiple Intelligences theory [18]. Thus, besides intelligence (IQ), we focused on other types of intelligences of the students, including verbal-linguistic, bodily-kinesthetic, and interpersonal intelligences.

Second, the Project adopted Renzulli’s (1978) three-ring conception of giftedness [2], which regards above average ability, high level of task commitment and creativity are three core elements for developing gifted behaviors of individuals. In Project GIFT, these three-ring elements were included in the Level 1 program with school-based enrichment curricula utilizing differentiated instructions as a universal design. Being one of the widely adopted curriculum models for gifted education across the world [19], it serves as a guiding reference to nurture creativity and promote the engagement of students through curriculum compacting, enrichment, differentiation, and acceleration in this project. 

Third, to capture different potential and abilities of students, we also developed a talent search database for students in each school based on the ideas of the Talent Search Model (TSM) [20]. Theoretically, TSM is a program model of overall talent development with an emphasis on acceleration options for advanced and high-ability learners [21]. However, TSM has also been adopted as a model for talent discovery and development [22]. It highlights the use of standardized procedures of screening, verification, and placement for the gifted and talented. Project GIFT took TSM into account and utilized the systemic approach in conducting universal screening and a data-informed approach for talent identification and search. Students with different potentials were identified by assessing their non-verbal logical reasoning, creativity, academic performances, multiple intelligences, gifted characteristics, general self-efficacy, psychological well-being, and satisfaction with life.

Fourth, in line with the significance of adopting the whole-school approach to offer quality educational services to students, Project Gift collaborated with the experimental schools to formulate their school-based talent development and gifted education policy in alignment with their major areas of concern in their school development plans. As such, a favorable school culture for gifted education and appropriate school policies and practices were developed to facilitate the implementation of Level 1 and Level 2 programs of school-based gifted education to students [23].

Fifth, an ecological approach [24] was adopted which emphasizes the impact of the interaction of inherent qualities of children and their environments on their growth and development. Given the importance of ecological environments such as schools and families on the development of children and adolescents, Project GIFT offers services and provisions to different school stakeholders, including school personnel, teachers, and parents besides students. 

**Figure 1:**
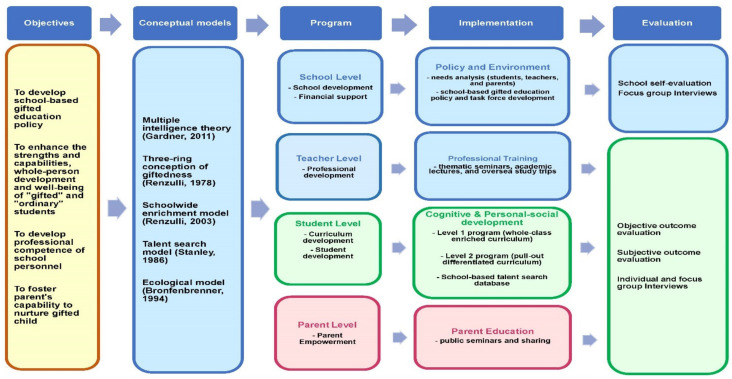
The intervention model of Project GIFT.

Based on above conceptual building blocks, Project GIFT developed an intervention model for school-based gifted education and talent development in alignment with the three-tier implementation model (i.e., Tier 1 and Tier 2 levels) of gifted education for Hong Kong schools. The service delivery model comprises four levels: school, teacher, student, and parent with six components of gifted education services and provisions, including curriculum development, student development, school development, professional development to teachers, financial support, and parent empowerment (Figure 1). 

**Figure 2:**
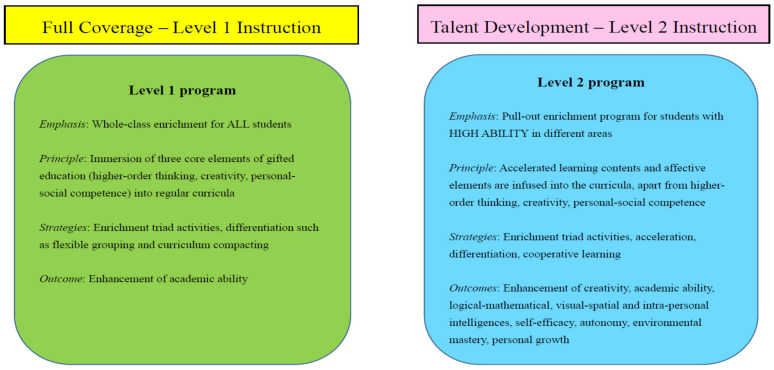
Level 1 and Level 2 programs.

Regarding curriculum development, two tiers (Level 1 and Level 2) programs were developed and implemented (Figure 2). The Level 1 program was a school-based whole-class teaching program in which higher-order thinking, creativity, and personal–social competence were infused into enriched and extended curricula of regular classrooms for all students [25]. In primary schools, the subjects involved were Chinese Language Education, English Language Education, Mathematics Education, and Science Education. In secondary schools, we focused on Mathematics Education and Science Education. According to Bloom’s (1956) taxonomy of educational objectives [26], learning objectives could be classified into six levels including knowledge, comprehension, application, analysis, synthesis, and evaluation. Of these six levels, analysis, synthesis, and evaluation were closely related to higher-order thinking. Therefore, enriched curricula were created by incorporating the tasks which promote students to differentiate facts, integrate different elements into a sound structure, and come up with judgments about the importance of concepts. 

Creativity is one of the constituents in Renzulli’s three-ring conception of giftedness. It is important to global development because it enriches the envision of analyses and helps resolve scientific questions in new and original ways [27]. As such, enriched curricula of this study were composed of various learning activities to encourage students to express their own ideas, such as divergent thinking and sensitivity to problems. 

Personal–social competence is conceptualized as one’s attitude towards one’s self (self-perception) and others (relationship with siblings, peers, parents, and elders), and one’s convictions, values, and concerns about society [28]. To foster students’ personal and social competencies, the curriculum was enriched by involving more collaborative learning activities. During the lessons, teachers encouraged students to respect and appreciate the opinions of others, to participate actively, to cordially cooperate with or to offer an assistance to others, and to share willingly the achievements with peers. 

After curriculum development, enriched curricula were implemented by utilizing enrichment activities of the Enrichment Triad in the Schoolwide Enrichment Model [29] and differentiation strategies such as flexible grouping and curriculum compacting. With reference to the Triad model, there are three kinds of enrichment experiences for students [19]. Type I enrichment is designed to expose students to a wide variety of disciplines and to stimulate new interests. Type II enrichment includes materials and methods designed to promote the development of thinking and feeling processes and it is usually provided to groups of students in their classroom or enrichment programs. Type III enrichment involves students who become interested in pursuing a self-selected area and are willing to commit the time necessary for advanced content acquisition and process training in which they assume the role of first-hand inquirer. The products can be completed by individuals or small groups of students and are always based on students’ interests. In Project GIFT, general exploratory learning activities (i.e., Type I enrichment) were used to expose students to the topics and stimulate their learning interest. Group activities (Type II enrichment) were given to students for training their creative thinking, problem-solving, critical thinking, and affective processes, for learning how-to-learn skills, skills in the use of advanced-level reference materials, and communication skills. Small-scale investigation of real problems (Type III enrichment) was used in some cases whenever appropriate. 

The Level 2 program is a school-based pull-out program in which differentiated curricula are designed for students with high ability in different areas. Students with high potential and abilities are identified based on the school-based talent search databases, examination results, performance and awards in competitions, as well as teacher-, parent-, and self-nominations. Students are selected to participate in pull-out programs which provide accelerated learning contents to students apart from the incorporation of higher-order thinking, creativity, and personal–social competence into the curricula. The learning content might be cross-subject with independent and group investigative tasks. The same as the Level 1 program, the curriculum is implemented by using enrichment activities of the Triad model in the Level 2 program. To facilitate the personal–social development of gifted students, affective elements and cooperative learning are incorporated into the curriculum. Students are offered opportunities to receive, respond, value, organize and internalize values for their affective development (Student development). Level 2 programs aim to strengthen the skills of high-ability students in leadership, emotional management, communication and collaboration, and independent research was constructed and implemented. For example, the program of “Photo-taking and Writing” was conducted to enhance students’ information technology, communication and collaboration skills. The program of “Little scientists” was used to promote investigative skills of students. Other Level 2 programs included “Extension of Pythagoras’ Theorem”, “English gifted class”, “Mock trial”, “Reading and creative writing class”, and “Micro:bit programming class”.

In addition to curriculum development, Project GIFT conducted onsite school visits and assisted schools in reviewing and refining their school-based gifted education and talent development policies. Further, the Project helped schools develop school-based talent search databases, which enables teachers to understand their students’ potential, interests, and learning abilities better. In the database, a talent portfolio with the assessment of academic ability, creativity, non-verbal logical reasoning, multiple intelligences, gifted characteristics, self-efficacy, psychological well-being, and satisfaction with life was created for each student. Accordingly, teachers can address diverse needs and characteristics of students, and design enriched curricula, differentiated pull-out programs, and teaching strategies (School development). Moreover, to advance teachers’ knowledge of gifted education and strategies in catering for students with high ability, Project GIFT organized different academic lectures and thematic seminars on gifted education, curriculum development, nurturance of creative and productive giftedness, affective education to the gifted, differentiation, and action research to teachers. It also facilitated local and overseas professional exchange through organizing cluster professional sharing sessions, overseas study trips, and presentations in foreign conferences (Professional development). 

To provide more resources, time and space for schools to develop and promote school-based gifted education, subsidies were given to experimental schools. Some schools were granted with HKD 10,000 to purchase equipment and resources for school-based gifted education while some schools were granted with HKD 330,000 for the employment of one additional teacher to reduce workload of the members of the Gifted Education Task Force and hence free the members to organize and conduct professional sharing activities (Financial support). Apart from supports offered to schools and teachers, Project GIFT stressed the importance of parent empowerment and home–school collaboration to nurture the potential of gifted and talented students. The Project organized seminars on cognitive and affective needs of gifted children, the parental role in nurturing the gifted, and home–school cooperation in gifted education (Parent empowerment). 

To understand the impact of the intervention, the two-group pretest–post-test design was adopted to assess the effectiveness of Project GIFT. In the experimental group, 20 primary and secondary schools in Hong Kong served as the project schools (N = 2453). Eight primary and secondary schools with similar characteristics were chosen as the control groups (N = 754). The general hypothesis was that students in the experimental groups at post-test (Level 1 and/or Level 2 programs) would have better outcomes than the students in the control groups after controlling the pretest measures (i.e., ANCOVAs).

## 2. Materials and Methods

### 2.1. Participants

A total of 20 experimental schools and 8 control schools in Hong Kong were recruited to participate in the Project based on purposive sampling. Experimental schools were chosen according to four main selection criteria: (a) schools demonstrating strong commitment and openness for gifted education and talent development; (b) schools admitting students with different academic achievement; (c) schools with a significant number of underprivileged students; and (d) schools from different school sponsoring bodies. Control schools were selected which matched the background of experimental schools (e.g., school banding, medium of instruction) and the demography of experimental students (e.g., grade level). At pretest, there were 2578 (response rate = 94.6%) and 771 students (response rate = 97.6%) in the experimental and control groups, respectively. At post-test, 2551 students in the experimental group (response rate = 93.6%) and 768 students in the control group (response rate = 97.2%) participated in the study. All students completed validated outcome measures of this study. Based on the matching information, we successfully matched the pretests and post-tests of 3207 students. The demographic information of the participants is shown in Table 1. The present Chi-square test results illustrated that experimental and control groups did not differ in the proportion of age group (*p* = 0.508), grade level (*p* = 0.179), and school type (*p* = 1.00). However, we cannot compare the sex distribution among the experimental and control groups because of a large number of missing responses to gender in the control group.

### 2.2. Instruments

In this study, validated measures on multiple intelligences, gifted characteristics, self-efficacy, psychological well-being, satisfaction with life, creativity, and non-verbal logical reasoning were utilized. Respondents were asked to indicate whether the items of the following validated scales accurately describe their experience along a 5-point scale with response options ranging from 1 (= not at all like me) to 5 (= very much like me). 

#### 2.2.1. Multiple Intelligences

The Student Multiple Intelligence Profile (SMIP) was used to examine the profile of multiple intelligences of students. It is composed of 24 items reflecting eight intelligences, namely, verbal–linguistic, musical, logical–mathematical, visual–spatial, bodily–kinesthetic, intrapersonal, interpersonal, and naturalistic intelligences [30]. SMIP was used in previous research with Chinese students and showed good psychometric properties (e.g., [30,31]). In this study, confirmatory factor analysis (CFA) supported the original eight-factor structure of SMIP at pretest (χ^2^ = 2298.75, df = 224, *p* < 0.001; NNFI = 0.96, CFI = 0.97, RMSEA = 0.057, SRMR = 0.065) and at post-test (χ^2^ = 2375.80, df = 224, *p* < 0.001; NNFI = 0.97, CFI = 0.98, RMSEA = 0.056, SRMR = 0.064). The composite reliability of the subscales ranged from 0.68 to 0.86 and from 0.73 to 0.90 at pretest and post-test, respectively. 

#### 2.2.2. Gifted Characteristics

The 12-item Gifted Characteristics Inventory (GCI) was developed by David Chan and Lai Kwan Chan in this study (Appendix A), who are scholars who have worked in gifted education for more than 30 years. It is used to assess personality characteristics of gifted students such as intense interests and active inquiry. Exploratory factor analysis (EFA) using the principal axis factoring method was used to explore the factor structure of GCI. The results revealed the unidimensional structure of GCI. The CFA results further validated the one-factor structure of GCI at pretest (χ^2^ = 724.39, df = 54, *p* < 0.001; NNFI = 0.97, CFI = 0.97, RMSEA = 0.065, SRMR = 0.037) and at post-test (χ^2^ = 860.11, df = 54, *p* < 0.001; NNFI = 0.97, CFI = 0.98, RMSEA = 0.071, SRMR = 0.035). The composite reliability of GCI was 0.86 and 0.90 at pretest and post-test, respectively.

#### 2.2.3. Self-Efficacy

The 10-item General Self-Efficacy Scale (GSE) was used to assess students’ optimistic self-beliefs in performing difficult tasks in different domains of human functioning [32]. Past research with Chinese samples has indicated that GSE possesses adequate psychometric properties (e.g., [32,33]). In this study, CFA results revealed that the data fitted the one-factor model well (at pretest: χ^2^ = 597.55, df = 35, *p* < 0.001; NNFI = 0.98, CFI = 0.98, RMSEA = 0.074, SRMR = 0.033; at post-test: χ^2^ = 1024.03, df = 35, *p* < 0.001; NNFI = 0.97, CFI = 0.97, RMSEA = 0.099, SRMR = 0.038). The composite reliability of GSE was 0.90 and 0.92 at pretest and post-test, respectively.

#### 2.2.4. Psychological Well-Being

The Psychological Well-Being Scale (PWBS) was used to measure the psychological well-being of students based on the positive psychology perspective [15]. It is composed of 24 items reflecting six dimensions including autonomy, environmental mastery, personal growth, positive relations with others, purpose in life, and self-acceptance. Previous research with Chinese students has shown that PWBS is valid and reliable [15]. The present CFA results supported the six-factor model of PWBS at pretest (χ^2^ = 1758.27, df = 237, *p* < 0.001; NNFI = 0.99, CFI = 0.99, RMSEA = 0.047, SRMR = 0.033) and at post-test (χ^2^ = 2163.93, df = 237, *p* < 0.001; NNFI = 0.99, CFI = 0.99, RMSEA = 0.053, SRMR = 0.036). The composite reliability of the subscales ranged from 0.76 to 0.84 and from 0.80 to 0.87 at pretest and post-test, respectively. 

#### 2.2.5. Life Satisfaction

The Satisfaction with Life Scale (SWLS) was used to measure the general life satisfaction of students [34]. SWLS is a 5-item scale which has been used in past research with Chinese samples and has shown adequate psychometric properties (e.g., [34,35]). The present CFA results supported the one-factor structure of SWLS at pretest (χ^2^ = 31.73, df = 4, *p* < 0.001; NNFI = 0.99, CFI = 1.00, RMSEA = 0.047, SRMR = 0.013) and at post-test (χ^2^ = 60.65, df = 4, *p* < 0.001; NNFI = 0.98, CFI = 0.99, RMSEA = 0.066, SRMR = 0.017). The composite reliability of SWLS was 0.81 and 0.85 at pretest and post-test, respectively.

Apart from above validated scales, participants were asked to complete two objective tests. First, 60-item Raven’s Standard Progressive Matrices and 36-item Raven’s Advanced Progressive Matrices were used to assess the non-verbal logical reasoning ability of primary students and secondary students, respectively [36]. Respondents were required to choose one of eight patterns that best solved the matrix. These Matrices, suited for group assessment, have been normed and standardized for students in Hong Kong [37]. They have been utilized to estimate general intelligence of Hong Kong students who are recruited to gifted programs [38]. Second, Wallach–Kogan tests on ideational fluency were used to assess creativity of students [39]. Ideational fluency is the tendency to generate all possible ideas and associations for familiar items. The tests on ideational fluency include instances, alternate uses, similarities, pattern meanings, and line meanings. In this study, the test on alternate uses was utilized [40]. Primary students were asked to list (by words and drawings) lots of different ways they could use an old book while secondary students were asked to list (by words and drawings) lots of different ways they could use a newspaper. Completion of the test was limited to 7 minutes. After completion of the test, the total number of distinct ideas were counted as the index of creativity.

### 2.3. Procedures

A quasi-experimental design was utilized to compare the experimental groups with the control group in this study. An introduction seminar about Project GIFT was opened to the principals and teachers of all Hong Kong primary and secondary schools in April 2017. Afterwards, an invitation letter accompanied with the outline of the Project were sent to the school principals. Among the 70 schools which responded to the Project, 20 schools were purposively selected as the experimental schools in August 2017. Written consent forms completely signed by students and parents to participate in the Project were obtained. Subsequently, students of the experimental groups completed the assessment at two different times between March 2018 and June 2019. At pretest, students completed intelligence and creativity tests, and the questionnaire. Students were required to write down their names, classes and class numbers as identifiers to match pre- and post-tests. At the same time, student’s age and examination results were obtained from school records. The assessment at post-test was the same as those at pretest except the intelligence test which had not been conducted repeatedly. The attrition rate was 1.1%. Eight control schools were chosen purposively in January 2019. Students of the control group completed same assessment as did the experimental students between January 2019 and June 2019. The kind of assessment and the time lag between two assessments were the same as those for the experimental students. The attrition rate was 0.4%.

### 2.4. Data Analysis

Only those students who had successful matching between pre- and post-tests were included in the data analysis. As the missing rate of the data was 7.8%, imputation was conducted to preserve the statistical power of the study [41]. Missing data at pretest was replaced by the post-test data of the same person (next-observation-carried-backward method) while missing data at post-test was substituted by the pretest data (last-observation-carried-forward method) [42]. These methods were preferable above mean substitution and regression methods to replace missing longitudinal data (see [43,44]). To assess for the impact of the intervention on each outcome variable, we used one-way ANCOVA to compare post-test scores between the experimental groups and control groups when controlling for pretest scores. In this study, we did not use the Bonferroni correction method to adjust *p*-values in multiple-testing process. This was because outcome variables were treated independently and only one analysis per outcome variable was performed (see [45]). Therefore, a difference between two means was regarded as statistically significant if the *p*-value was less than 0.05. The effect size was measured by Cohen’s d with 0.2 as small, 0.5 as medium, and 0.8 as large [46]. A negative effect size indicated a reduction in the mean score of the outcome while a positive effect size showed an increase. One-way ANCOVA was conducted using SPSS 26.0.

## 3. Results

Descriptive statistics, Cronbach’s alphas and mean inter-item correlations of all outcome variables in each group (Level 1 program only, both Level 1 and Level 2 programs, and control group) are illustrated in Table 2. All measures were reliable (Cronbach’s alphas ranged from 0.63 to 0.92) (see [47,48]). One-way ANCOVA was used to test whether students in the experimental groups at post-test (Level 1 program only and/or both Level 1 and 2 programs) achieved better outcomes than students in the control group after controlling for the pretest measures. 

**Figure 3:**
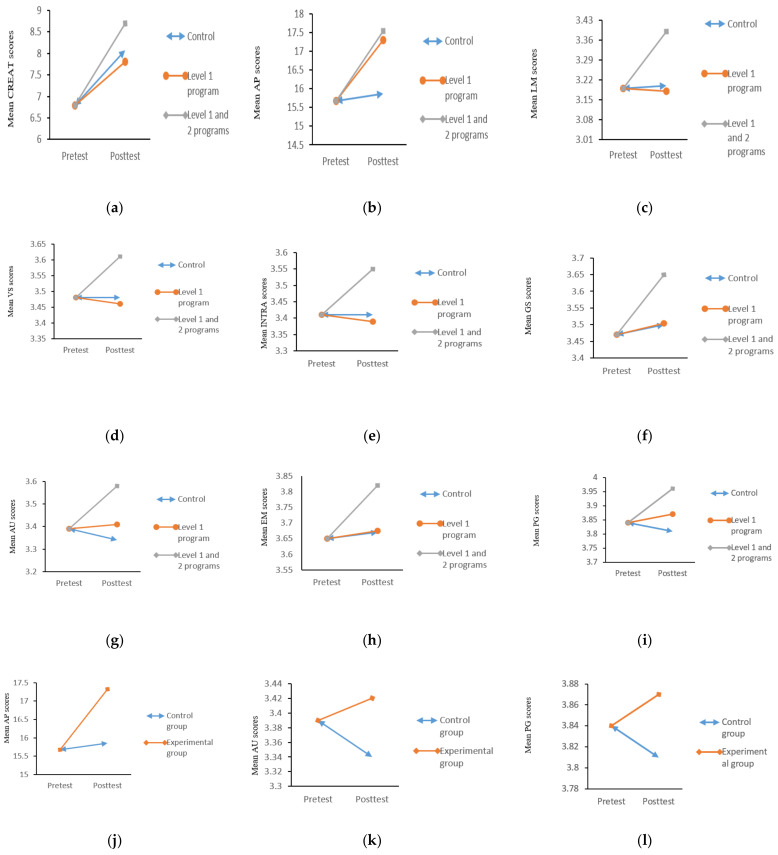
(**a**–**l**) Graphs showing the difference in outcome variables at post-test from one-way ANCOVA amongst Level 1 program, Level 1 and 2 programs, and the Control (**a**–**i**). Graphs showing the difference in outcome variables at post-test from one-way ANCOVA between experimental and control groups (**j**–**l**). (**a**). CREAT (creativity), Note: F(2,3178) = 5.30, *p* =0.005, (**b**). AP (Academic performance), Note: F(2,3177) = 40.42, *p* = 0.000, (**c**). LM (logical–mathematical intelligence), Note: F(2,3179) = 5.06, *p* = 0.006, (**d**). VS (visual–spatial intelligence), Note: F(2,3179) = 3.08, *p* = 0.046, (**e**) INTRA (intrapersonal intelligence), Note: F(2,3179) = 3.90, *p* = 0.020, (**f**). GS (self-efficacy), Note: F(2,3179) = 3.84, *p* = 0.022, (**g**). AU (autonomy), Note: F(2,3179) = 5.98, *p* = 0.003, (**h**). EM (environmental mastery), Note: F(2,3179) = 3.13, *p* = 0.044, (**i**). PG (personal growth), Note: F(2,3179) = 3.56, *p* = 0.029, (**j**). AP (academic performance), Note: F(1,3178) = 80.30, *p* = 0.000, (**k**). AU (autonomy), Note: F(1,3180) = 5.24, *p* = 0.022, (**l**). PG (personal growth), Note: F(1,3180) = 4.33, *p* = 0.037.

With reference to the three groups (students joining Level 1 Program, students joining both Level 1 plus Level 2 program, and control group), ANCOVA results revealed significant difference(s) in post-test scores of creativity, F(2,3178) = 5.30, *p* < 0.01; academic performance, F(2,3177) = 40.42, *p* < 0.001; logical–mathematical intelligence, F(2,3179) = 5.06, *p* < 0.01; visual–spatial intelligence, F(2,3179) = 3.08, *p* < 0.05; intrapersonal intelligence, F(2,3179) = 3.90, *p* < 0.05; self-efficacy, F(2,3179) = 3.84, *p* < 0.05; autonomy, F(2,3179) = 5.98, *p* < 0.01; environmental mastery, F(2,3179) = 3.13, *p* < 0.05; and personal growth, F(2,3179) = 3.56, *p* < 0.05 among three groups (Level 1 program only, both Level 1 and 2 programs, and the control). The difference(s) in each outcome variable is graphically presented in Figure 3a–i. 

Table 3 illustrates the results of the ANCOVA post hoc analysis. The adjusted mean score of creativity in both Level 1 and 2 programs was significantly higher than those of the Level 1 program only (d = 0.23) and those of the control group (d = 0.16). Regarding academic performance, the adjusted mean score of both Level 1 and 2 programs was greater than those of the control group (d = 0.44). Similarly, the adjusted mean score of Level 1 program only was more than those of the control group (d = 0.38). Besides, both Level 1 and 2 programs showed higher adjusted mean score in logical–mathematical intelligence than Level 1 program only (d = 0.24) and the control group (d = 0.23); higher adjusted mean score in intrapersonal intelligence than Level 1 program only (d = 0.18) and the control group (d = 0.17); higher adjusted mean score in self-efficacy than Level 1 program only (d = 0.26) and the control group (d = 0.25); higher adjusted mean score in autonomy than Level 1 program only (d = 0.19) and the control group (d = 0.30), higher adjusted mean score in environmental mastery than Level 1 program only (d = 0.17) and the control group (d = 0.19), as well as higher adjusted mean score in personal growth than Level 1 program only (d = 0.10) and the control group (d = 0.19). In addition, both Level 1 and 2 programs showed higher adjusted mean score in visual–spatial intelligence than those of Level 1 program only (d = 0.17). 

Regarding the overall effect of the Project on students (i.e., combined experimental groups versus controls), the outcomes of the overall sample in the experimental group and the control sample were compared. Descriptive statistics, Cronbach’s alphas and mean inter-item correlations of all outcome variables in the experimental and control groups are illustrated in Table 4. All measures achieved an acceptable level of reliability (Cronbach’s alphas ranged from 0.64 to 0.92). 

The ANCOVA results revealed significant difference(s) in post-test scores of academic performance, F(1,3178) = 80.30, *p* < 0.001; autonomy, F(1,3180) = 5.24, *p* < 0.05, and personal growth, F(1,3180) = 4.33, *p* < 0.05. The difference(s) in each outcome variable are graphically presented in Figure 3j–l.

The results of the post hoc analysis in Table 5 revealed that both Level 1 and 2 programs have higher adjusted mean scores in academic performance (d = 0.38), autonomy (d = 0.09), and personal growth (d = 0.07) than those of the control group. 

## 4. Discussion

In view of the paucity of research on gifted education frameworks for delivering learning opportunities and services to the gifted and talented, and limited studies on research-validated instructional practices in gifted education in Hong Kong, Project GIFT was launched to respond to this deficiency in the literature. The Project served as a pioneering school-based gifted education program which provided Level 1 and Level 2 programs for primary and secondary students in Hong Kong. One of the strengths of this study is that a large sample was recruited which responded to the call of researchers for using a large and representative sample to increase the credibility of the findings in gifted studies [49]. Second, both primary and secondary school students were recruited in this study. This responds to the urge of educators to examine the impact of gifted education on students across different grade levels (e.g., [4,50]). Third, this study utilized an experimental design with control groups to investigate the intervention effect of the gifted education program on students. This strengthens the rigor of past gifted studies which have commonly used descriptive and correlational methodologies [51]. Fourth, this study utilized locally validated scales to perform holistic investigation of a broad array of cognitive (e.g., academic achievement, intelligence), affective (e.g., psychological well-being, self-efficacy), and personality effects (e.g., gifted characteristics) on students. This outperforms past gifted studies which have only focused on either cognitive or socio-emotional outcomes of students (e.g., [4,52]). Fifth, as there is scant research on school-based gifted education in Chinese contexts, this study adds on the literature of gifted education in non-Western countries such as Hong Kong. 

The present study reported the objective outcome evaluation findings of the Project. The findings are very positive and consistent with original expectations. The Level 1 program was designed and implemented to all regular students. The results showed that students of the Level 1 program had higher academic achievement in language, mathematics, and science subjects than students of the control groups after participating in the program. These changes might be attributed to the immersion of higher-order thinking skills and creativity into the enriched curricula, which could assist students in learning and enhance students’ motivation to study. The results are aligned with [5], which revealed an increase in reading performance after immersing higher-order thinking skills and differentiated instruction into classroom teaching, and [53] which indicated the enhancement of students’ motivation to learn via teachers’ creativity-fostering practices in regular classrooms. 

The benefits of pull-out programs outside the regular classroom (Level 2 programs) are also shown. In fact, the results are very encouraging and impressive. They revealed that students participating in both Level 1 and Level 2 programs had higher level of creativity, academic performance, self-efficacy, logical–mathematical intelligence, visual–spatial intelligence, intrapersonal intelligence, autonomy, environmental mastery, and personal growth than control students, with low-to-medium effect sizes. Thus, apart from the enhancement of student learning as conducted in the Level 1 program, affective and personal–social development of students were also promoted after participating in Level 2 programs, such as an increase in self-confidence to solve problems, the ability to control learning and the sense of personal growth. As such, participation in both Level 1 and Level 2 programs incrementally facilitated cognitive, affective and personal–social development of students. These changes might be attributed to the incorporation of affective elements into enriched and differentiated curricula, apart from the immersion of three core elements of gifted education (higher-order thinking, creativity, and personal–social competence). The positive effects of Level 2 programs on gifted students are aligned with empirical findings that have supported the utility of pull-out programs on cognitive, affective and psychosocial development of gifted students (e.g., [52,54,55]). Compared pull-out programs to within-classroom provisions, [56] illustrated that pull-out gifted education programs had greater impact on students. It is because students in pull-out programs receive lessons at their own pace, more focused instruction on higher-level cognitive activities and individual support for extension. As such, students from pull-out groups displayed more positive attitudes toward the work that they did.

In summary, the beneficial effects of Level 1 and Level 2 programs on students were empirically supported. Nevertheless, the interpersonal relationship of experimental students was not enhanced, although the element of personal-social competence such as cooperative learning and collaborative activities were immersed into enriched curricula and differentiated pull-out programs. As stated by [57], teachers usually wrongly perceive that their pupils could learn social skills incidentally through their interaction in group activities. In fact, social skills should be taught to students prior to asking students to engage in cooperative tasks. In future studies, we should further reflect on how social skills training can be incorporated into gifted education programs in addition to collaborative learning activities. 

Regarding the findings from the whole sample of experimental students, students of the combined experimental groups had higher level of academic performance, autonomy and personal growth than control students, with low-to-medium effect sizes. These results are promising which indicate that school-based gifted education could promote cognitive and affective development of students. 

## 5. Implications

There are several theoretical and practical implications of the findings as far as the development and implementation of school-based gifted education are concerned. First, in conjunction with other evidence in Western contexts (e.g., [58,59,60]), the present study offers empirical support for the positive impact of different instructional practices on students such as curriculum compacting, differentiation and enrichment triad activities. Second, the findings of this study also suggest that the three-tier implementation model of gifted education could serve not only as a valid and viable model for the systematic delivery of learning opportunities and services to the gifted and talented, but also as the basis for quality teaching [61]. As such, this study is an important contribution to local literature of gifted education. Third, the RTI model has been developed originally to offer systematic intervention to students with learning difficulties [62]. This study supports the fact that the three-tier implementation model of gifted education (particularly the Tier 1 and Tier 2 programs), developed from the RTI framework, assist both regular and gifted students in a holistic development beyond academic improvement. Fourth, the school-based gifted education program of this study was developed based on Renzulli’s three-ring conception of giftedness. Apart from adoption of the basic tenets of this conception, we have advanced the research on Renzulli’s three-ring conception in terms of methodological rigor. As stated by [63], the strength of Renzulli’s enrichment triad model is its focus on discerning the interests of all students and providing encouragement for their development through enrichment activities. Nevertheless, the weakness of this model is the lack of school-based assessment procedures that guide decisions about a broader range of enrichment, acceleration and other types of program options for gifted students with different needs. This study assessed the performance of students in various domains at the beginning of a school year. Subsequently, the assessment results were used as the foundation for teachers to adopt different enrichment and differentiated instructional strategies in classroom teaching. After adopting different teaching strategies, teachers re-assessed students and adjusted their teaching strategies accordingly. Hence, the present study enriches Renzulli’s enrichment triad model with school-based assessment practices and brings about many positive student outcomes.

Practically, in view of the dearth of evaluation studies that inform gifted education practices in Asian countries [64], the research-based gifted education programs developed in this study could act as exemplars for researchers to create multi-level intervention to the gifted and talented in other Asian and international contexts. As stated by [65,66], giftedness is culturally defined and hence it is important to take cultural contexts into account to understand different gifted education programs and policies in different countries. The findings of this study support the generalizability of the enrichment models across cultures. In view of the huge population of children and adolescents in China, research on gifted education programs in Chinese contexts undoubtedly enriches the designation of gifted education interventions across different cultures. Indeed, the three-tier model adopted in this study and Level 1 and Level 2 enrichment programs are commonly adopted in other parts of the world (e.g., [5,52,54]). However, there are few studies focusing on the Level 1 and Level 2 programs simultaneously. Hence, the findings can provide support for such models (i.e., theoretical significance). The models could serve as an important reference for colleagues in other parts of the world to adopt (i.e., practical significance). Moreover, by adopting the multidimensional conception of giftedness, we have supported the schools to establish their own talent-search databases for students based on students’ logical reasoning ability, academic ability, creativity, and psychological profiles. The information from the database would help teachers not only select appropriate enrichment and differentiated instruction for students in classroom teaching, but also choose students to participate in appropriate pull-out programs. As the optimal match between a gifted student’s demonstrated abilities, achievements and interests and their educational provisions is a prerequisite for students to maximize their potential [22], the database could serve as a template for other schools to develop their own talent search databases. In addition, the Project stresses the influence of different stakeholders such as school personnel, teachers, and parents on students from the ecological perspective. As such, continued school support and training for teachers and parents were provided so as to bring about positive student outcomes. The positive attainment of the Project offers an insight into different success factors for the implementation of school-based gifted education such as school development and professional training for teachers. 

## 6. Limitations

Several limitations of this study need to be mentioned. First, the results should be treated as preliminary because this study adopted a quasi-experimental design to examine the impact of Level 1 and Level 2 programs on students, with participants not randomly allocated to the experimental and control groups. Although the findings are positive with the control of pretest scores in the analyses, future research should replicate this study using randomized controlled trials in multiple sites conducted by different teams of independent researchers. Second, the findings of this study only illustrate the objective outcome evaluation of the Project. However, other evaluation methods such as subjective outcome evaluation and qualitative focus groups [67,68] would be utilized to triangulate the data and enhance the credibility and validity of the findings. Third, as self-reported measures were utilized to collect data in this study, there was a risk of common method variance bias which posed the threat to the validity of the findings [69]. The correlations between variables might be artificially inflated because of the common method used to collect data. Therefore, future research might use the measures of different sources. For instance, teacher rating could be utilized to assess interpersonal relationship and autonomy of students. Fourth, as this study are confined to Hong Kong school students, the generalizability of the findings to students in other Chinese communities is not knowable at this stage. Future research should replicate the study in other Chinese contexts. This would help support the external validity and lend credibility to the conclusions of the present study. Fifth, since there were only two time points (i.e., pretest and post-test) in this study, only the short-term effects of the intervention were able to be assessed. Hence, future research should collect more longitudinal data to enable researchers to understand the long-term impact of the intervention. Last, only primary and secondary schools in Hong Kong were recruited in this study. Even though the size of the sample can be regarded as respectable, inclusion of more schools with diverse background characteristics such as kindergartens and universities would be useful. 

## 7. Conclusions

The present study addressed several conceptual and methodological limitations in the scientific literature on the development and implementation of gifted education programs for students. Conceptually, we proposed an integrated model based on the three-tier implementation model of gifted education to develop pioneer Level 1 and Level 2 programs for implementation. Methodologically, we recruited large samples of Hong Kong primary and secondary school students and utilized validated measures in a ground-breaking quasi-experimental study. In line with our expectations, this study revealed that students in the experimental groups showed positive changes after joining the program(s), with greater beneficial effects for students participating in both Level 1 and Level 2 programs. As such, the benefits of the Level 1 and Level 2 programs in enhancing the holistic development of students are illustrated.

## Figures and Tables

**Table 1:** Demographic information of the participants in different groups.

Groups	Experimental Groups			Control Group (N = 754)
	Overall (N = 2453)	Level 1 Program Only (N = 2277)	Both Level 1 and 2 Programs (N = 176)	
Gender				
Male	1328	1223	105	241
Female	1124	1053	71	93
Missing	1	1	0	420
Age (Mean, SD)	(10.36, 1.50)	(10.33, 1.50)	(10.88, 1.46)	(10.89, 1.84)
Missing	8	8	0	47
Grade				
Primary 3	579	555	24	0
Primary 4	682	658	24	174
Primary 5	728	651	77	380
Primary 6	0	0	0	0
Secondary 1	346	306	40	0
Secondary 2	117	106	11	135
Secondary 3	0	0	0	65
Missing	1	1	0	0
School type				
Primary schools	15	15	13	6
Secondary schools	5	5	5	2

**Table 2:** Descriptive statistics and reliabilities of all outcome variables in Level 1 program only, both Level 1 and 2 programs, and control group.

	Experimental Groups								Control Group			
	Level 1 Program Only				Both Level 1 and 2 Programs							
	Pretest		Post-Test		Pretest		Post-Test		Pretest		Post-Test	
Variable	M (SD)	α (IIC)	M (SD)	α (IIC)	M (SD)	α (IIC)	M (SD)	α (IIC)	M (SD)	α (IIC)	M (SD)	α (IIC)
CREAT	6.42 (4.18)	-	7.56 (4.61)	-	7.99 (4.08)	-	9.52 (5.24)	-	7.62 (4.59)	-	8.64 (5.00)	-
AP	14.55 (8.91)	-	16.20 (9.67)	-	18.43 (9.28)	-	20.22 (9.96)	-	18.44 (10.82)	-	18.55 (10.84)	-
MI												
VI	3.05 (0.88)	0.65 (0.38)	3.09 (0.88)	0.69 (0.42)	3.26 (0.89)	0.74 (0.48)	3.34 (0.91)	0.74 (0.49)	3.00 (0.90)	0.68 (0.41)	3.08 (0.92)	0.72 (0.45)
MU	3.44 (1.08)	0.78 (0.55)	3.48 (1.08)	0.83 (0.63)	3.53 (1.03)	0.80 (0.58)	3.55 (1.02)	0.83 (0.62)	3.51 (1.07)	0.80 (0.57)	3.49 (1.10)	0.85 (0.65)
LM	3.14 (1.03)	0.71 (0.45)	3.16 (1.05)	0.79 (0.56)	3.55 (0.99)	0.77 (0.52)	3.61 (1.01)	0.79 (0.55)	3.23 (1.08)	0.78 (0.53)	3.22 (1.05)	0.78 (0.54)
VS	3.46 (0.97)	0.66 (0.39)	3.45 (0.95)	0.71 (0.45)	3.62 (0.87)	0.64 (0.37)	3.69 (0.89)	0.77 (0.54)	3.52 (0.94)	0.67 (0.41)	3.50 (0.94)	0.69 (0.43)
BK	3.43 (0.95)	0.64 (0.39)	3.40 (0.99)	0.72 (0.47)	3.41 (0.96)	0.63 (0.38)	3.43 (1.06)	0.82 (0.61)	3.42 (0.97)	0.66 (0.40)	3.42 (0.97)	0.70 (0.45)
INTRA	3.40 (0.89)	0.70 (0.44)	3.39 (0.90)	0.76 (0.51)	3.53 (0.81)	0.66 (0.40)	3.62 (0.82)	0.73 (0.48)	3.39 (0.88)	0.69 (0.43)	3.40 (0.91)	0.75 (0.50)
INTER	3.80 (0.88)	0.77 (0.53)	3.75 (0.90)	0.82 (0.61)	3.83 (0.88)	0.82 (0.60)	3.79 (0.83)	0.83 (0.62)	3.79 (0.87)	0.77 (0.53)	3.77 (0.88)	0.78 (0.54)
NAT	3.02 (1.19)	0.85 (0.66)	3.00 (1.18)	0.89 (0.73)	2.98 (1.16)	0.88 (0.72)	2.97 (1.16)	0.90 (0.75)	2.93 (1.20)	0.86 (0.66)	2.92 (1.22)	0.89 (0.72)
GC	3.91 (0.69)	0.86 (0.35)	3.90 (0.72)	0.90 (0.43)	4.13 (0.57)	0.83 (0.29)	4.12 (0.64)	0.90 (0.42)	3.96 (0.67)	0.86 (0.35)	3.91 (0.71)	0.88 (0.40)
GSE	3.44 (0.81)	0.90 (0.46)	3.49 (0.82)	0.92 (0.53)	3.64 (0.72)	0.89 (0.45)	3.74 (0.71)	0.91 (0.49)	3.50 (0.79)	0.90 (0.46)	3.52 (0.81)	0.91 (0.51)
PWB												
AU	3.37 (0.94)	0.78 (0.47)	3.40 (0.95)	0.83 (0.55)	3.59 (0.85)	0.76 (0.44)	3.67 (0.93)	0.86 (0.60)	3.41 (0.96)	0.80 (0.50)	3.35 (0.96)	0.82 (0.54)
EM	3.64 (0.91)	0.83 (0.55)	3.66 (0.91)	0.87 (0.63)	3.81 (0.82)	0.82 (0.53)	3.89 (0.76)	0.82 (0.54)	3.65 (0.90)	0.83 (0.55)	3.67 (0.90)	0.85 (0.59)
PG	3.81 (0.88)	0.80 (0.51)	3.85 (0.85)	0.84 (0.57)	4.13 (0.69)	0.69 (0.36)	4.11 (0.78)	0.83 (0.56)	3.86 (0.87)	0.80 (0.50)	3.82 (0.88)	0.83 (0.54)
PR	3.53 (0.89)	0.74 (0.42)	3.54 (0.91)	0.79 (0.49)	3.55 (0.86)	0.76 (0.45)	3.59 (0.91)	0.83 (0.55)	3.52 (0.94)	0.77 (0.46)	3.49 (0.93)	0.79 (0.48)
PL	3.67 (0.93)	0.83 (0.54)	3.66 (0.95)	0.87 (0.63)	3.87 (0.87)	0.83 (0.56)	3.83 (0.85)	0.86 (0.61)	3.68 (0.96)	0.84 (0.58)	3.61 (0.97)	0.86 (0.61)
SA	3.68 (0.90)	0.79 (0.49)	3.66 (0.91)	0.84 (0.56)	3.82 (0.80)	0.75 (0.43)	3.83 (0.80)	0.82 (0.54)	3.67 (0.92)	0.81 (0.51)	3.63 (0.92)	0.82 (0.53)
SWL	3.45 (0.92)	0.79 (0.45)	3.43 (0.94)	0.84 (0.53)	3.44 (0.93)	0.83 (0.52)	3.44 (0.94)	0.86 (0.56)	3.36 (0.95)	0.81 (0.47)	3.37 (0.98)	0.84 (0.53)

Note. M = mean; SD = standard deviation; IIC = mean inter-item correlation; CREAT = creativity; AP = academic performance; MI = multiple intelligence; VI = verbal–linguistic intelligence; MU = musical intelligence; LM = logical–mathematical intelligence; VS = visual–spatial intelligence; BK = bodily–kinesthetic intelligence; INTRA = intrapersonal intelligence; INTER = interpersonal intelligence; NAT = naturalistic intelligence; GC = gifted characteristics; GSE = general self-efficacy; PWB = psychological well-being; AU = autonomy; EM = environmental mastery; PG = personal growth; PR = positive relations with others; PL = purpose in life; SA = self-acceptance; SWL = satisfaction with life.

**Table 3:** Results of one-way ANCOVA on all outcome variables of students in Level 1 program only, both Level 1 and 2 programs, and control.

Variable		Level 1 Program Only	Both Level 1 And 2 Programs	Control Group	*F* Value	*df*	*p*-Value	R^2^ (%)
	Pretest	Post-Test	Post-Test	Post-Test				
	AM	AM(SE)	AM(SE)	AM(SE)				
CREAT	6.79	7.81 (0.08)	8.69 (0.28)	8.07 (0.14)	5.30	2, 3178	0.005	38.7
AP	15.68	17.30 (0.08)	17.53 (0.29)	15.86 (0.14)	40.42	2, 3177	0.000	85.3
MI								
VI	3.05	3.09 (0.02)	3.23 (0.06)	3.11 (0.03)	2.82	2, 3179	0.060	29.9
MU	3.46	3.50 (0.02)	3.50 (0.07)	3.46 (0.03)	0.62	2, 3179	0.536	37.0
LM	3.19	3.18 (0.02)	3.39 (0.06)	3.20 (0.03)	5.06	2, 3179	0.006	35.0
VS	3.48	3.46 (0.02)	3.61 (0.06)	3.48 (0.03)	3.08	2, 3179	0.046	29.2
BK	3.42	3.40 (0.02)	3.44 (0.06)	3.42 (0.03)	0.55	2, 3179	0.578	37.9
INTRA	3.41	3.39 (0.02)	3.55 (0.06)	3.41 (0.03)	3.90	2, 3179	0.020	28.3
INTER	3.80	3.75 (0.02)	3.77 (0.06)	3.77 (0.03)	0.45	2, 3179	0.641	32.2
NAT	3.00	2.98 (0.02)	2.98 (0.08)	2.96 (0.04)	0.16	2, 3179	0.849	30.6
GC	3.94	3.91 (0.01)	4.00 (0.04)	3.89 (0.02)	2.41	2, 3179	0.090	33.9
GSE	3.47	3.50 (0.01)	3.65 (0.05)	3.50 (0.02)	3.84	2, 3179	0.022	32.5
PWB								
AU	3.39	3.41 (0.02)	3.58 (0.06)	3.34 (0.03)	5.98	2, 3179	0.003	25.2
EM	3.65	3.67 (0.02)	3.82 (0.06)	3.67 (0.03)	3.13	2, 3179	0.044	24.0
PG	3.84	3.87 (0.02)	3.96 (0.06)	3.81 (0.03)	3.56	2, 3179	0.029	26.6
PR	3.53	3.54 (0.02)	3.57 (0.06)	3.49 (0.03)	1.18	2, 3179	0.308	30.6
PL	3.69	3.67 (0.02)	3.73 (0.06)	3.61 (0.03)	1.81	2, 3178	0.164	29.8
SA	3.69	3.66 (0.02)	3.76 (0.06)	3.64 (0.03)	1.86	2, 3178	0.156	28.0
SWL	3.43	3.42 (0.02)	3.43 (0.06)	3.40 (0.03)	0.29	2, 3177	0.747	25.5

Note. AM = adjusted mean; CREAT = creativity; AP = academic performance; MI = multiple intelligence; VI = verbal–linguistic intelligence; MU = musical intelligence; LM = logical–mathematical intelligence; VS = visual–spatial intelligence; BK = bodily–kinesthetic intelligence; INTRA = intrapersonal intelligence; INTER = interpersonal intelligence; NAT = naturalistic intelligence; GC = gifted characteristics; GSE = general self-efficacy; PWB = psychological well-being; AU = autonomy; EM = environmental mastery; PG = personal growth; PR = positive relations with others; PL = purpose in life; SA = self-acceptance; SWL = satisfaction with life.

**Table 4:** Descriptive statistics and reliabilities of all outcome variables in the combined experimental groups and the control groups.

Variable	Combined Experimental Groups				Control Groups			
	Pretest		Post-Test		Pretest		Post-Test	
	M (SD)	α (IIC)	M (SD)	α (IIC)	M (SD)	α (IIC)	M (SD)	α (IIC)
CREAT	6.53 (4.19)	-	7.70 (4.69)	-	7.62 (4.59)	-	8.64 (5.00)	-
AP	14.83 (8.99)	-	16.49 (9.74)	-	18.44 (10.82)	-	18.55 (10.84)	-
MI								
VI	3.06 (0.88)	0.66 (0.39)	3.11 (0.89)	0.69 (0.43)	3.00 (0.90)	0.68 (0.41)	3.08 (0.92)	0.72 (0.45)
MU	3.44 (1.07)	0.79 (0.55)	3.49 (1.08)	0.83 (0.62)	3.51 (1.07)	0.80 (0.57)	3.49 (1.10)	0.85 (0.65)
LM	3.17 (1.03)	0.72 (0.46)	3.19 (1.05)	0.80 (0.56)	3.23 (1.08)	0.78 (0.53)	3.22 (1.05)	0.78 (0.54)
VS	3.47 (0.96)	0.66 (0.39)	3.46 (0.95)	0.71 (0.45)	3.52 (0.94)	0.67 (0.41)	3.50 (0.94)	0.69 (0.43)
BK	3.43 (0.95)	0.64 (0.38)	3.40 (0.99)	0.72 (0.48)	3.42 (0.97)	0.66 (0.40)	3.42 (0.97)	0.70 (0.45)
INTRA	3.41 (0.88)	0.70 (0.44)	3.40 (0.89)	0.76 (0.51)	3.39 (0.88)	0.69 (0.43)	3.40 (0.91)	0.75 (0.50)
INTER	3.80 (0.88)	0.77 (0.53)	3.75 (0.90)	0.82 (0.61)	3.79 (0.87)	0.77 (0.53)	3.77 (0.88)	0.78 (0.54)
NAT	3.02 (1.19)	0.85 (0.66)	2.99 (1.18)	0.89 (0.73)	2.93 (1.20)	0.86 (0.66)	2.92 (1.22)	0.89 (0.72)
GC	3.93 (0.68)	0.86 (0.35)	3.91 (0.72)	0.90 (0.43)	3.96 (0.67)	0.86 (0.35)	3.91 (0.71)	0.88 (0.40)
GSE	3.46 (0.80)	0.90 (0.46)	3.51 (0.81)	0.92 (0.53)	3.50 (0.79)	0.90 (0.46)	3.52 (0.81)	0.91 (0.51)
PWB								
AU	3.39 (0.94)	0.78 (0.47)	3.42 (0.95)	0.83 (0.56)	3.41 (0.96)	0.80 (0.50)	3.35 (0.96)	0.82 (0.54)
EM	3.65 (0.91)	0.83 (0.55)	3.68 (0.90)	0.87 (0.62)	3.65 (0.90)	0.83 (0.55)	3.67 (0.90)	0.85 (0.59)
PG	3.84 (0.87)	0.80 (0.50)	3.87 (0.85)	0.84 (0.57)	3.86 (0.87)	0.80 (0.50)	3.82 (0.88)	0.83 (0.54)
PR	3.53 (0.89)	0.74 (0.42)	3.54 (0.91)	0.80 (0.50)	3.52 (0.94)	0.77 (0.46)	3.49 (0.93)	0.79 (0.48)
PL	3.69 (0.93)	0.83 (0.54)	3.67 (0.94)	0.87 (0.63)	3.68 (0.96)	0.84 (0.58)	3.61 (0.97)	0.86 (0.61)
SA	3.69 (0.89)	0.79 (0.48)	3.67 (0.90)	0.84 (0.56)	3.67 (0.92)	0.81 (0.51)	3.63 (0.92)	0.82 (0.53)
SWL	3.44 (0.92)	0.79 (0.45)	3.43 (0.94)	0.84 (0.53)	3.36 (0.95)	0.81 (0.47)	3.37 (0.98)	0.84 (0.53)

Note. M = mean; SD = standard deviation; IIC = mean inter-item correlation; CREAT = creativity; AP = academic performance; MI = multiple intelligence; VI = verbal–linguistic intelligence; MU = musical intelligence; LM = logical–mathematical intelligence; VS = visual–spatial intelligence; BK = bodily–kinesthetic intelligence; INTRA = intrapersonal intelligence; INTER = interpersonal intelligence; NAT = naturalistic intelligence; GC = gifted characteristics; GSE = general self-efficacy; PWB = psychological well-being; AU = autonomy; EM = environmental mastery; PG = personal growth; PR = positive relations with others; PL = purpose in life; SA = self-acceptance; SWL = satisfaction with life.

**Table 5:** Results of one-way ANCOVA on all outcome variables of students in the combined experimental groups and the control groups.

Variable		Combined Experimental Group	Control Group	*F* Value	*df*	*p*-Value	R^2^ (%)
	Pretest	Post-Test	Post-Test				
	AM	AM(SE)	AM(SE)				
CREAT	6.79	7.87 (0.08)	8.07 (0.14)	1.53	1, 3179	0.216	38.5
AP	15.68	17.32 (0.08)	15.86 (0.14)	80.30	1, 3178	0.000	85.3
MI							
VI	3.05	3.10 (0.02)	3.11 (0.03)	0.08	1, 3180	0.772	29.8
MU	3.46	3.50 (0.02)	3.46 (0.03)	1.24	1, 3180	0.265	37.0
LM	3.19	3.20 (0.02)	3.20 (0.03)	0.00	1, 3180	0.974	34.8
VS	3.48	3.47 (0.02)	3.48 (0.03)	0.14	1, 3180	0.710	29.1
BK	3.42	3.40 (0.02)	3.42 (0.03)	0.50	1, 3180	0.481	37.9
INTRA	3.41	3.40 (0.02)	3.41 (0.03)	0.16	1, 3180	0.691	28.1
INTER	3.80	3.75 (0.02)	3.77 (0.03)	0.65	1, 3180	0.420	32.2
NAT	3.00	2.98 (0.02)	2.96 (0.04)	0.32	1, 3180	0.572	30.6
GC	3.94	3.92 (0.01)	3.89 (0.02)	0.96	1, 3180	0.327	33.8
GSE	3.47	3.51 (0.01)	3.50 (0.03)	0.15	1, 3180	0.697	32.4
PWB							
AU	3.39	3.42 (0.02)	3.34 (0.03)	5.24	1, 3180	0.022	25.0
EM	3.65	3.68 (0.02)	3.67 (0.03)	0.08	1, 3180	0.772	23.8
PG	3.84	3.87 (0.02)	3.81 (0.03)	4.33	1, 3180	0.037	26.5
PR	3.53	3.54 (0.02)	3.49 (0.03)	1.98	1, 3180	0.160	30.6
PL	3.69	3.67 (0.02)	3.61 (0.03)	2.70	1, 3179	0.100	29.8
SA	3.69	3.67 (0.02)	3.64 (0.03)	0.86	1, 3179	0.353	28.0
SWL	3.43	3.43 (0.02)	3.40 (0.03)	0.58	1, 3178	0.448	25.5

Note. AM = adjusted mean; CREAT = creativity; AP = academic performance; MI = multiple intelligence; VI = verbal–linguistic intelligence; MU = musical intelligence; LM = logical–mathematical intelligence; VS = visual–spatial intelligence; BK = bodily–kinesthetic intelligence; INTRA = intrapersonal intelligence; INTER = interpersonal intelligence; NAT = naturalistic intelligence; GC = gifted characteristics; GSE = general self-efficacy; PWB = psychological well-being; AU = autonomy; EM = environmental mastery; PG = personal growth; PR = positive relations with others; PL = purpose in life; SA = self-acceptance; SWL = satisfaction with life.

## Data Availability

The data presented in this study are only available on request from the corresponding author.

## Appendix A. Gifted Characteristics Inventory

**Items**
1. I concentrate on things that I am interested in.
2. I have my own opinion on many issues.
3. If I am doing things of my interest, I will not give up easily when facing difficulties or failures.
4. I enjoy leisure reading.
5. I have keen interest in some topics.
6. I interact with people in a fair and just manner.
7. I am willing to spend a great deal of time and energy on what I am interested in.
8. I strive for excellence in whatever I do.
9. When I have difficulties, I try to solve it by myself first.
10. I actively search for information related to my interests.
11. I like exploring new things.
12. I work hard to keep myself up in some areas.
Note. This scale was developed by David Chan and Lai-kwan Chan. David Chan and Lai-kwan Chan are Program Founders and Honorary Program Director in the Program for the Gifted and Talented in Faculty of Education, The Chinese University of Hong Kong, respectively. They are scholars who have been working in gifted education for more than 30 years. In Project GIFT, David Chan was an Honorary Advisor and Lai-kwan Chan was a Co-Chief Principal Investigator of the project. She was the Project Operation Leader and Budget Holder from December 2016 to December 2018.

## References

[1] Hernández-Torrano D., Saranli A.G. (2015). A cross-cultural perspective about the implementation and adaptation process of the schoolwide enrichment model: The importance of talent development in a global world. Gift. Educ. Int..

[2] Renzulli J.S. (1978). What makes giftedness? Re-examining a definition. Phi Delta Kappan.

[3] Rogers K.B., Assouline S.G., Colangelo N., VanTassel-Baska J., Lupkowski-Shoplik A. (2015). The academic, socialization, and psychological effects of acceleration: Research synthesis. A Nation Empowered: Evidence Trumps the Excuses Holding Back America’s Brightest Students.

[4] Kim M. (2016). A meta-analysis of the effects of enrichment programs on gifted students. Gift. Child Q..

[5] Shaunessy-Dedrick E., Evans L., Ferron J., Lindo M. (2015). Effects of differentiated reading on elementary students’ reading comprehension and attitudes toward reading. Gift. Child Q..

[6] Steenbergen-Hu S., Makel M.C., Olszewski-Kubilius P. (2016). What one hundred years of research says about the effects of ability grouping and acceleration on k-12 students’ academic achievement: Findings of two second-order meta- analyses. Rev. Educ. Res..

[7] James A.L. (2018). What Are the Effects of Curriculum Compacting on Students’ Ability to Use Higher Order Thinking?. Ph.D. Thesis.

[8] Bicknell B. (2008). Gifted students and the role of mathematics competitions. Aust. Prim. Math. Classr..

[9] Johnsen S.K., Parker S.L., Farah Y.N. (2015). Providing services for students with gifts and talents within a response-to-intervention framework. Teach. Except. Child..

[10] Raben K., Brogan J., Dunham M., Contreras Bloomdahl S. (2019). Response to intervention (RTI) and changes in special education categorization. Except. Educ. Int..

[11] Poon-McBrayer K.F. (2018). Practicing response-to-intervention model: A case of leadership practices. Int. J. Whole Sch..

[12] Cheung R.S.H., Hui A.N.N., Cheung A.C.K. (2020). Gifted education in Hong Kong: A school-based support program catering to learner diversity. ECNU Rev. Educ..

[13] Chan D.W. (2004). Multiple intelligences of Chinese gifted students in Hong Kong: *Perspectives from students, parents, teachers, and peers*. Roeper Rev..

[14] Chan D.W., Chan L.K., Chau A. (2009). Judging drawing abilities of Hong Kong Chinese gifted students: Could nonexperts make expert-like judgments?. Gift. Child Q..

[15] Chan D.W., Chan L.K., Sun X. (2019). Developing a brief version of Ryff’s scale to assess the psychological well-being of adolescents in Hong Kong. Eur. J. Psychol. Assess..

[16] Kwan A.C.K., Yuen M. (2013). “Mathematics in the workplace”: A pilot enrichment programme for mathematically talented primary students in Hong Kong. Gift. Talent. Int..

[17] Brown E.F. (2012). Is response to intervention and gifted assessment compatible?. J. Psychoeduc. Assess..

[18] Gardner H. (2011). Frames of Mind: The Theory of Multiple Intelligences.

[19] Reis S.M., Peters P.M. (2021). Research on the schoolwide enrichment model: Four decades of insights, innovation, and evolution. Gift. Educ. Int..

[20] Stanley J.C., Benbow C.P., Sternberg R.J., Davidson J.E. (1986). Youths who reason exceptionally well mathematically. Conceptions of Giftedness.

[21] Chan D.W., Wright J.D. (2015). Education for the gifted and talented. International Encyclopedia of the Social and Behavioural Sciences.

[22] Lupkowski-Shoplik A., Beneow C.P., Assouline S.G., Brody L.E., Colangelo N., Davis G.A. (2003). Talent searches: Meeting the needs of academically talented youth. Handbook of Gifted Education.

[23] Education Bureau (2014). Operation Guide on the Whole School Approach to Integrated Education. https://sense.edb.gov.hk/uploads/page/integrated-education/guidelines/ie_guide_en.pdf.

[24] Bronfenbrenner U., Gauvain M., Cole M. (1994). Ecological models of human development. Readings on the Development of Children.

[25] Education Bureau (2015). Gifted Education in Hong Kong Book 1: Learning about Gifted Education. https://www.edb.gov.hk/attachment/tc/curriculum-development/major-level-of-du/gifted/resources_and_support/ge_resource_bank/files/Policy/GE_info_booklet/GE_info_booklet_1_eng.pdf.

[26] Bloom B.S. (1956). Taxonomy of Educational Objectives: The Classification of Educational Goals Handbook 1: Cognitive Domain.

[27] Renzulli J.S., Reis S.M. (2014). The Schoolwide Enrichment Model: A How-to-Guide for Educational Excellence.

[28] Education Bureau (2018). Guidelines on School-based Gifted Development Programmes. [Archive] Appendices. https://www.edb.gov.hk/attachment/en/curriculum-development/major-level-of-edu/gifted/guidelines-on-school-based-gifted-development-programmes/app_1-eng.pdf.

[29] Renzulli J.S. (2003). The schoolwide enrichment model: An overview of the theoretical and organizational rationale. Gift. Educ. Int..

[30] Chan D.W. (2003). Multiple intelligences and perceived self-efficacy among Chinese secondary school teachers in Hong Kong. Educ. Psychol..

[31] Chan D.W. (2006). Perceived multiple intelligences among male and female Chinese gifted students in Hong Kong: The structure of the student multiple intelligences profile. Gift. Child Q..

[32] Chan D.W. (2002). Stress, self-efficacy, social support, and psychological distress among prospective Chinese teachers in Hong Kong. Educ. Psychol..

[33] Zhang J.X., Schwarzer R. (1995). Measuring optimistic self-beliefs: A Chinese adaptation of the General Self-Efficacy Scale. Psychologia.

[34] Chan D.W. (2012). Life satisfaction, happiness, and the growth mindset of healthy and unhealthy perfectionists among Hong Kong Chinese gifted students. Roeper Rev..

[35] Chan D.W. (2009). Orientations to happiness and subjective well-being among Chinese prospective and in-service teachers in Hong Kong. Educ. Psychol..

[36] Raven J.C., Court J.H., Raven J. (1987). A Manual for Raven’s Progressive Matrices and Vocabulary Tests.

[37] Hong Kong Education Department (1986). Hong Kong Supplement to Guide to the Standard Progressive Matrices.

[38] Chan D.W. (2000). Identifying gifted and talented students in Hong Kong. Roeper Rev..

[39] Wallach M.A., Kogan N. (1965). Modes of Thinking in Young Children.

[40] Chan D.W., Cheung P.C., Lau S., Wu W.Y.H., Kwong J.M.L., Li W.L. (2001). Assessing ideational fluency in primary students in Hong Kong. Creat. Res. J..

[41] Tsikriktsis N. (2005). A review of techniques for treating missing data in OM survey research. J. Oper. Manag..

[42] Tsang H.W.H., Cheung W.M., Chan A.H.L., Fung K.M.T., Leung A.Y., Au D.W.H. (2013). A pilot evaluation on a stress management programme using a combined approach of cognitive behavioural therapy (CBT) and complementary and alternative medicine (CAM) for elementary school teachers. Stress Health.

[43] Engels J.M., Diehr P. (2003). Imputation of missing longitudinal data: A comparison of methods. J. Clin. Epidemiol..

[44] Twisk J., de Vente W. (2002). Attrition in longitudinal studies: How to deal with missing data. J. Clin. Epidemiol..

[45] Cabin R.J., Mitchell R.J. (2000). To bonferroni or not to bonferroni: When and how are the questions. Bull. Ecol. Soc. Am..

[46] Cohen J. (1988). Statistical Power Analysis for the Behavioral Sciences.

[47] Bhatnagar R., Kim J., Many J.E. (2014). Candidate surveys on program evaluation: Examining instrument reliability, validity and program effectiveness. Am. J. Educ. Res..

[48] Morgan G.A., Leech N.L., Gloeckner G.W., Barrett K.C. (2013). IBM SPSS for Introductory Statistics: Use and Interpretation.

[49] Makel M.C., Smith K.N., Miller E.M., Peters S.J., McBee M.T. (2020). Collaboration in giftedness and talent development research. J. Educ. Gift..

[50] Kettler T., Oveross M.E., Bishop J.C. (2017). Gifted education in preschool: Perceived barriers and benefits of program development. J. Res. Child. Educ..

[51] Plucker J.A., Callahan C.M. (2014). Research on giftedness and gifted education: Status of the field and considerations for the future. Except. Child..

[52] Gubbels J., Segers E., Verhoeven L. (2014). Cognitive, socioemotional, and attitudinal effects of triarchic enrichment program for gifted children. J. Educ. Gift..

[53] Regier P., Savic M. (2020). How teaching to foster mathematical creativity may impact student self-efficacy for proving. J. Math. Behav..

[54] Golle J., Zettler I., Rose N., Trautwein U., Hasselhorn M., Nagengast B. (2018). Effectiveness of a “Grass Roots” statewide enrichment program for gifted elementary school children. J. Res. Educ. Eff..

[55] Van der Meulen R.T., van der Bruggen C.O., Spilt J.L., Verouden J., Berkhout M., Bögels S.M. (2013). The pullout program day a week school for gifted children: Effects on social-emotional and academic functioning. Child Youth Care Forum.

[56] Dimitriadis C. (2012). Provision for mathematically gifted children in primary schools: An investigation of four different methods of organizational provision. Educ. Rev..

[57] Chan K.W. (2014). Cooperative learning in a Hong Kong primary school: Perceptions, problems and accommodation. Intercult. Educ..

[58] Demir S. (2021). Effects of learning style based differentiated activities on gifted students’ creativity. J. Educ. Gift. Young Sci..

[59] Joli N.S., Kamarulzaman M.H., Rashid S.A., Hazir N.M., Simin N., Hissam F.A.B. (2020). Curriculum compacting: Differentiating statistics syllabus according to the readiness levels of gifted students. Int. J. Educ. Pedagog..

[60] Milan L., Reis S.M. (2021). The implementation of the schoolwide enrichment model in Italian schools. Int. J. Talent Dev. Creat..

[61] Peterson M. (2007). Whole Schooling Consortium: Eight Principles of Whole Schooling. http://www.wholeschooling.net/WS/WS%208%20Principles.html.

[62] Siegel L.S. (2020). Early identification and intervention to prevent reading failure: A response to intervention (RTI) initiative. Educ. Dev. Psychol..

[63] Olszewski-Kubilius P. (1999). A critique of Renzulli’s theory into practice models for gifted learners. J. Educ. Gift..

[64] Chan D.W., Pfeiffer S.I., Shaunessy-Dedrick E., Foley-Nicpon M. (2018). Gifted education in Asia. APA Handbook of Giftedness and Talent.

[65] Sternberg R.J. (2007). Cultural concepts of giftedness. Roeper Rev..

[66] Zhang Z. (2017). Gifted education in China. Cogent Educ..

[67] Shek D.T.L., Ng C.S.M. (2009). Subjective outcome evaluation of the project P.A.T.H.S. (Secondary 2 program): Views of the program participants. Sci. World J..

[68] Shek D.T.L., Ng C.S.M. (2009). Qualitative evaluation of the project P.A.T.H.S.: Findings based on focus groups with student participants. Sci. World J..

[69] Reio T.G. (2010). The threat of common method variance bias to theory building. Hum. Resour. Dev. Rev..

